# *Orostachys japonicus* induce p53‐dependent cell cycle arrest through the MAPK signaling pathway in OVCAR‐3 human ovarian cancer cells

**DOI:** 10.1002/fsn3.836

**Published:** 2018-10-25

**Authors:** Kyung‐Sun Lee, Suhng‐Wook Kim, Hyeong‐Seon Lee

**Affiliations:** ^1^ Department of Integrated Biomedical and Life Sciences Graduate School Korea University Seoul Korea; ^2^ Department of Biomedical Laboratory Science Jungwon University Goesan Chungbuk Korea

**Keywords:** apoptosis, cell cycle, *Orostachys japonicus*, ovarian cancer cells

## Abstract

*Orostachys japonicus* (*O. japonicus*) is utilized as a traditional medicine for patients with various diseases. This study investigated the effect of the ethyl acetate fraction from *O. japonicus* extract (OJE) on the growth inhibition of OVCAR‐3 human ovarian cancer cells demonstrated to inhibit cell growth and arrest the cell cycle in OVCAR‐3 cells by blocking the sub‐G1 phase and decreasing cyclin E1/CDK2 expression. Cell cycle arrest was connected to the increased expression of the cell cycle regulating factors p53 and p21. Apoptosis was initiated through the intrinsic pathway by up‐regulating the expression of the Bcl‐2/Bax ratio and down‐regulating the expression of pro‐caspase‐3. Furthermore, OJE treatment elicited p‐p38 activation and p‐ERK1/2 inhibition. In conclusion, our results demonstrated that OJE reduced the growth of OVCAR‐3 human ovarian cancer cells mediated by arrest of the cell cycle and regulation of MAPK signaling pathways.

## 1 INTRODUCTION

Human ovarian cancer is a major cause of gynecological cancer death in women. Surgical castration and chemotherapy are mainly used in cancer treatments, including ovarian cancer (Jemal et al., 2008; Shirali et al., 2013). Treated patients often experience reoccurrence within a short period of time that is resistant to chemotherapy. As a result, the 5‐year survival rate is known <30% (Aletti, Gallenberg, Cliby, Jatoi, & Hartmann, 2007). Therefore, there is an increasing need for effective agents from medicinal products against ovarian cancer.

*Orostachys japonicus* (Crassulaceae), a perennial herb employed as a medicinal plant, has been used as a folk treatment for inflammatory, febrile, hemostatic, antidote, and variable cancers (Park, Han, Park, Choi, & Choi, 2005; Ryu, Lee, Lee, & Lee, 2012). *Orostachys japonicus* is reported to contain 16 types of flavonoid contents. They have been confirmed to have antioxidant activity using DPPH assay (Lee et al., 2011). However, the exact activity of its physiological effects and the cell signaling pathways involved remain unknown. In our laboratory, the powder of *O. japonicus* was fractionated with organic solvents (EtOH, hexane, DCM, EtOAc, BuOH, and H_2_O). We studied the anti‐cancer activity of *O. japonicus* in human gastric and hepatoma cancer cells. We determined that its ability to suppress cancer cell proliferation is mediated through an apoptotic mechanism. Among the *O. japonicus* extracts, EtOAc fraction showed the highest anticancer activity (Lee et al., 2014; Ryu et al., 2012). To our knowledge, there are no reports on anti‐cancer activity of the ethyl acetate fraction from *O. japonicus* extract (OJE) in human ovarian cancer cell lines. In this study, we investigated the effect of OJE on cell proliferation as well as its apoptotic pathway and cell cycle progression in the OVCAR‐3 human ovarian cancer cell line.

## 2 MATERIALS AND METHODS

### 2.1 Cell culture and reagents

OVCAR‐3 human ovarian cancer cells were obtained from the Korean Cell Line Bank (KCLB, Seoul, Korea). Cells were cultured in Roswell Park Memorial Institute (RPMI) 1,640 medium (Gibco/Invitrogen, USA) added with 10% fetal bovine serum (FBS; HyClone, USA), penicillin (100 U/ml), and streptomycin (100 μg/ml) at 37°C in a 5% CO_2_. The cells were sub‐cultured every 2–3 days at 1:5 split ratios. Primary antibodies against phospho‐ERF1/2, phospho‐p38, phosphor‐JNK, and GAPDH were purchased from Cell Signaling Technology (Beverly, USA). Secondary antibodies, an Annexing V‐FITC assay kit and cell cycle assay kit were purchased from BD Pharmingen^™^ (BD Biosciences, USA).

### 2.2 Preparation of OJE fraction from *O. japonicus*

Dried *O. japonicus* powder was supplied by Geobugiwasong Ltd. (Miryang, Korea). The ethyl acetate (EtOAc) fraction from *O. japonicus* was fractioned using a solvent, as described by our team (Lee et al., 2014; Ryu et al., 2012). The EtOAc fraction was concentrated by evaporation at 40°C to achieve dryness, and stored in dimethyl sulfoxide (DMSO) at −20°C.

### 2.3 GC‐MS analysis

Component analysis of the EtOAc fraction (OJE) has previously been described by our team (Lee et al., 2014; Ryu et al., 2012).

### 2.4 Cell viability assay

Cell viability was determined with a CellTiter 96 AQueous One Solution Cell Proliferation Assay Kit (Promega Corporation, Madison, WI, USA) according to the manual. OVCAR‐3 cells were incubated with serial concentrations (0, 12.5, 25, 50 μg/ml) of OJE for 24 hr. After incubation, 10 μl of MTS solution was added to the well and incubated for 3 hr. The absorbance in the wells was measured at 490 nm using a FilterMax F5 Multi‐Mode microplate reader (Molecular Devices, USA).

### 2.5 Quantification of apoptosis by flow cytometry

OVCAR‐3 cells were treated with OJE for 24 hr and harvested with 0.25% trypsin‐EDTA treatment. The apoptotic cells were detected using 10 μl of annexin V‐FITC and 5 μl of propidium iodine (PI) for 15 min in the dark (BD Biosciences, USA) and then analyzed with a FACSCalibur flow cytometer (BD Biosciences, USA). For each condition, populations of 1 × 10^4^ cells were determined in each cytometry experiment.

### 2.6 Cell cycle analysis

Cells (5 × 10^5^/ml) were plated in six‐well plates followed by treatment with OJE for 48 hr. The cell cycle phase was assayed by DNA fragment staining with PI solution using a cell cycle phase detection kit (BD Bioscience, USA). Cells were determined by FACSCalibur flow cytometry (BD Biosciences, USA).

### 2.7 Detection of apoptotic body by DAPI staining

The apoptotic bodies were stained using the 1 μg/ml DAPI solution (Vector Laboratories, USA) according to the manufacturer's instructions. Cells were treated with OJE fraction for 24 hr. After incubation, the cells were washed with cold PBS and then fixed in cold 4% paraformaldehyde for 30 min. Apoptotic bodies were dyed blue and fixed with mounting medium. After staining, cells were analyzed using fluorescence microscopy on AMG (Washington, USA).

### 2.8 RNA extraction and Reverse Transcription PCR

Cells were treated with different concentrations (0, 12.5, 25, 50 μg/ml) of OJE for 24 hr. Total RNA was isolated using the Trizol reagent (Invitrogen, USA). The concentration and purity of the RNA were measured by a FilterMax F5 Multi‐Mode microplate reader (Molecular Devices, USA). cDNA was synthesized using 1 μg of total RNA per 20 μl of reaction mixture using AccuPower RT PreMix reagent for the reverse transcription (Bioneer, Korea). Target gene duplication was performed using specific oligonucleotide primers of right and left in the PCR system. The primer sequences and conditions used in the PCRs are listed in Table 1. The PCR products were electrophoresed on agarose gels and stained using ethidium bromide (EtBr). The bands were determined and visualized using the Davinch‐Chemi^™^ imaging system (Davinch‐K, Korea).

**Table 1:** Primers for RT‐PCR

Gene	Sequence of primers		Size of products (bp)
CDK2	Sense	3′‐GCCCTAATCTCACCCTCTCC	211
	Antisense	3′‐AAGGGTGGTGGAGGCTAACT	
CyclinE1	Sense	3′‐TACCCAAACTCAACGTGCAA	473
	Antisense	3′‐AGGGGACTTAAACGCCACTT	
p21	Sense	3′‐TTAGCAGCGGAACAAGGAGT	225
	Antisense	3′‐GCCGAGAGAAAACAGTCCAG	
p53	Sense	3′‐GGCCCACTTCACCGTACTAA	485
	Antisense	3′‐AAGCGAGACCCAGTCTCAAA	
Bax	Sense	3′‐TTTGCTTCAGGGTTTCATCC	246
	Antisense	3′‐CAGTTGAAGTTGCCGTCAGA	
Bcl‐2	Sense	3′‐GGATGCCTTTGTGGAACTGT	457
	Antisense	3′‐GGTGCTTGGCAATTAGTGGT	
Pro‐caspase‐3	Sense	3′‐TTTTTCAGAGGGGATCGTTG	269
	Antisense	3′‐TCAAGCTTGTCGGCATACTG	
GAPDH	Sense	3′‐GTCAGTGGTGGACCTGACCT	420
	Antisense	3′‐AGGGGTCTACATGGCAACTG	

### 2.9 SDS‐PAGE and Western Blot analysis

OVCAR‐3 cells were treated with OJE for appropriate times. The cells rinsed twice with PBS and lysed using cell lysis buffer (Cell Signaling Technology, USA) containing a protease inhibitor cocktail in six‐well plates (Roche Diagnostics Ltd., Germany). The total concentration of protein was measured using a bicinchoninic acid (BCA) protein assay kit (Thermo Scientific, IL). To quantify the expression of the target protein, the sample was subjected to 8%–10% sodium dodecyl sulfate (SDS)‐polyacrylamide gel electrophoresis (PAGE) and transferred to polyvinylidene fluoride (PVDF) membranes. The membrane was blocked with 5% BSA, incubated with primary antibody (all antibodies were diluted at 1:1,000 in 3% BSA) for 24 hr at 4°C and incubated with horseradish peroxidase (HRP)‐conjugated secondary antibody (the antibody was diluted at 1:10,000 in 3% BSA) for 2.5 hr. The protein bands on the membrane were visualized using enhanced chemiluminescense (ECL) solution (Santa Cruz Biotechnology, USA), and signals were detected using the Davinch‐Chemi^™^ imaging system (Davinch‐K, Korea).

### 2.10 Statistical analysis

The results are expressed as mean ± standard deviation (*SD*) and analyzed by SPSS version 22.0 (SPSS Inc., USA). *p* < 0.05 was considered significantly.

## 3 RESULTS AND DISCUSSION

In this study, the intracellular signaling pathways of OJE‐induced apoptosis in OVCAR‐3 cells were investigated. *Orostachys japonicus* was extracted sequentially by organic solvents, including ethyl alcohol (EtOH), n‐hexane (Hex), dichloromethane (DCM), ethyl acetate (EtOAc), n‐butanol (BuOH), and water (H_2_O). Among these, the EtOAc fraction was the most active against OVCAR‐3 cancer cells. This fraction was analyzed using a GC‐MS system following the measurement methods detailed in our study. As a result, the following three peaks were identified: “gallic acid (4.24%), kaempferol (6.81%), and quercetin (5.08%)” (Lee et al., 2014; Ryu et al., 2012). Various studies have revealed that kaempferol and quercetin lead to apoptosis using the natural products in various human cancer cells (Lee, Szcsepanski, & Lee, 2008; Yoshida et al., 2008). However, many *O. japonicus* ingredients have not yet been identified and warrant further study to determine their various physiological activities.

### 3.1 OJE inhibits cell proliferation in OVCAR‐3 cells

**Figure 1:**
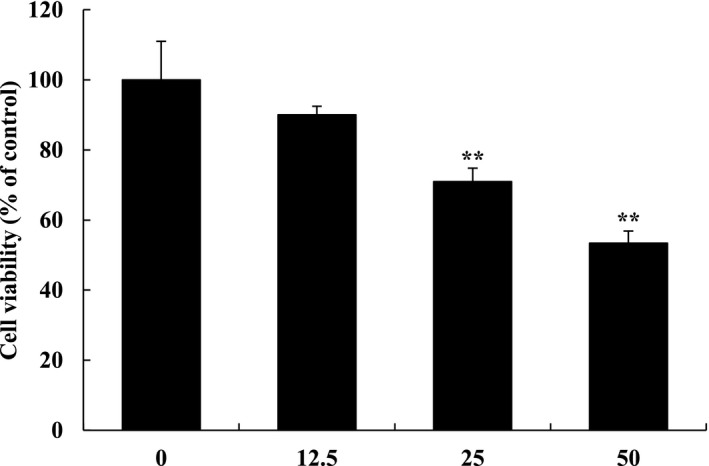
Growth inhibition effect of *Orostachys japonicus* extract (OJE) on OVCAR‐3 cells. OVCAR‐3 cells were incubated with OJE (0, 12.5, 25, 50 μg/ml) for 24 hr and the cell viabilities were determined by MTS assay. The values are expressed as the mean ± *SD*. **Significantly different from control at *p* < 0.01

An anticancer effect commonly involves mechanisms that induce apoptosis and abnormal cell growth inhibition. The suppression of abnormally proliferating cancer cells is an important screening result factor that proves the anticancer activity of drugs (Li et al., 2015). To confirm the effects of OJE on cell viability, we used the MTS assay. Treatment with OJE sequentially decreased cell viability in a concentration‐dependent manner. OVCAR‐3 cancer cells revealed cytotoxic effects of nearly 50% at a relatively high concentration (50 μg/ml). These results are similar to the effects of our previous studies on AGS human gastric cancer cells and HepG2 human hepatoma cancer cells at high concentration (100 μg/ml). Therefore, OJE can effectively induce OVCAR‐3 cancer cell death, even at half the concentrations of those observed in previous studies Figure 1.

### 3.2 OJE induces apoptosis in OVCAR‐3 cells

**Figure 2:**
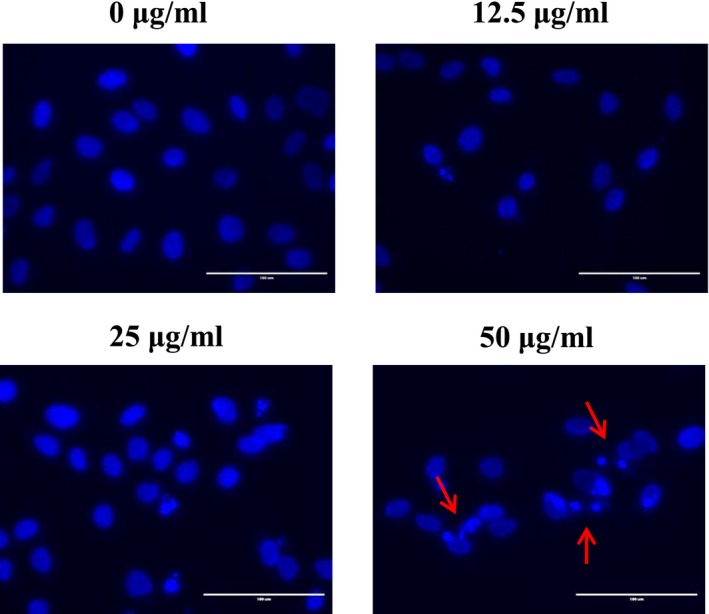
Effect of *Orostachys japonicus* extract (OJE) on apoptosis in the OVCAR‐3 cells. OVCAR‐3 cells were treated with OJE (0, 12.5, 25, 50 μg/ml) for 12 hr and then stained with DAPI solution. Apoptotic bodies were observed under the fluorescence microscopy

**Figure 3:**
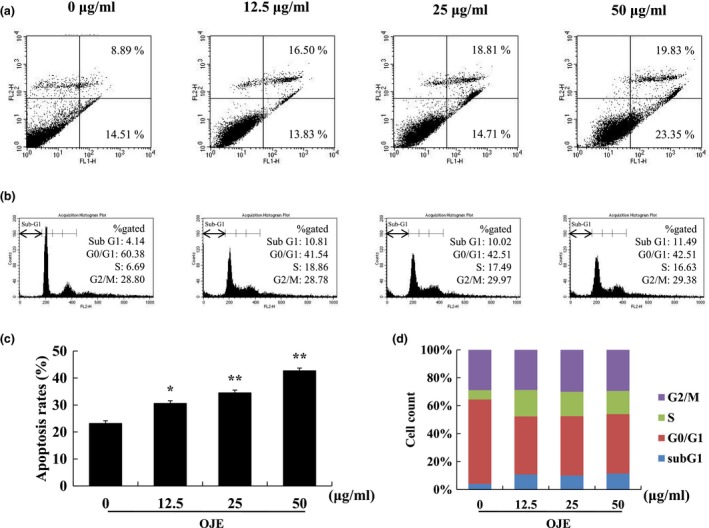
*Orostachys japonicus* extract (OJE) induces apoptosis and cell cycle arrest in the OVCAR‐3 cells. OVCAR‐3 cells were treated with OJE (0, 12.5, 25, 50 μg/ml) for 24 hr. The cells were assessed using Annexin V‐FITC/PI (a,c) and cell cycle flow cytometric (b,d) assay kit. The apoptotic cells were determined by flow cytometry

Programmed apoptosis cell death is an organized and regular process that maintains homeostasis in the body. It causes various phenomena, such as cellular shrinkage, chromatin condensation, blebbing, nuclear fragmentation, and formation of apoptotic bodies. Numerous variations are considered important products for the identification of cancer cell death (Evan & Vousden, 2001; Muppidi, Porter, & Siegel, 2004; Rello et al., 2005). To observe whether OJE generates apoptotic bodies, the nuclear fragmentations of OVCAR‐3 cancer cells have been imaged using fluorescence microscopy by DAPI staining (Figure 2a). Compared with untreated cells, OJE‐treated ovarian cancer cells distinctly exhibited numerous apoptotic cells with condensed or fragmented nuclei at high concentration (50 μg/ml). To evaluate the general effect of apoptosis, cells were stained with Annexin V‐FITC and PI, and then the cell population stages were analyzed using flow cytometry. The early or late apoptotic cells were classified as quadrants, which are presented in the lower‐right (Annexin V^+^/PI^−^) and upper‐right (Annexin V^+^/PI^+^) quadrants (Fu, Ye, Lee, Rankin, & Chen, 2017). OJE significantly induced the early and late apoptosis stages in a concentration‐dependent manner (Figure 3a). The rates of total apoptosis increased from 23.4% in the control cells to 30.35%, 33.52%, and 43.18% in OJE‐treated cells, respectively (Figure 3c).

### 3.3 OJE induces cell cycle arrest

Cell proliferation inhibition is closely related to cell cycle regulation in anti‐cancer mechanisms (Xie & Yang, 2014). Cell overgrowth is caused by a cell cycle checkpoint disorder. An abnormal cell cycle checkpoint is an intrinsic cancer property. It is used to study selective mechanisms for a range of cancer therapies (Gabrielli, Brooks, & Pavey, 2012). The cell cycle process is basically carried out in the order of the G1, S, and G2/M phases. When apoptosis is stimulated, the cell cycle process can be stopped at several stages (Stefanowicz‐Hajduk, Sparzak‐Stefanowska, Krauze‐Baranowska, & Ochocka, 2016). Based on this, the effect of OJE on the cell cycle phase of OVCAR‐3 cells was determined by flow cytometry. As shown in Figure 3(b,d), treatment with OJE effectively blocked cell cycle progression at the sub‐G1 phase. Thus, the cell density at the sub‐G1 significantly increased in a concentration‐dependent manner. The down‐regulation of cyclin E1/CDK2 expression arrested the G1/S phase of the cell cycle process (Singh, Banerjee, Acosta, Lillard, & Singh, 2017). Based on the result that OJE‐induced G1 arrest, we evaluated the effect of OJE on cell cycle regulatory mRNA expression. The levels of CyclinE1/CDK2 mRNA were significantly down‐regulated in the OJE‐treated OVCAR‐3 cells (Figure 3a).

**Figure 4:**
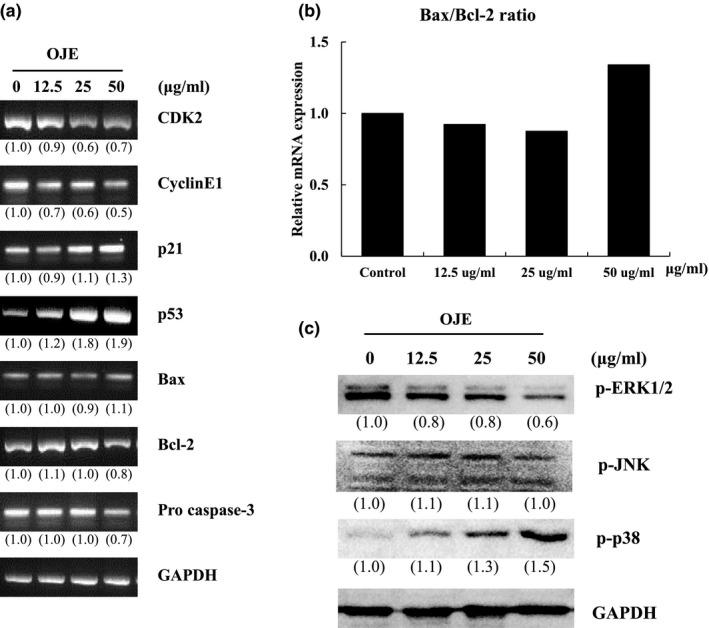
Effect of *Orostachys japonicus* extract (OJE) on apoptosis and cell cycle‐related mRNA and protein levels. OVCAR‐3 cells were treated with OJE (0, 12.5, 25, 50 μg/ml) for 1 or 24 hr. (a) Expression of CDK2, CyclinE1, p21, p53, Bax, Bcl‐2 and pro‐caspase‐3 were analyzed by RT‐PCR. (b) The ratio of Bas to Bcl‐2 was calculated. (c) Expression of MAPK signaling proteins was examined by western blotting assay

P53 is a tumor suppressor gene in cell cycle progression regulation during programmed cell death (Fan, Ma, Liu, Zheng, & Huang, 2014; Malumbres, 2014). The modulation of cell cycle‐related genes by p53 activation arrests the cell cycle checkpoint at the G1/S or G2/M phase. The p21 gene is activated by the p53 transcription and involved in the major cell cycle arrest function through cyclinE1/CDK2 suppression (Chen et al., 2016).To determine the cell cycle regulating factors in OJE‐treated OVCAR‐3 cells, we evaluated the p53 and p21 mRNA levels. The p53 and p21 mRNA levels gradually increased in a dose‐dependent manner in OJE‐treated OVCAR‐3 cells (Figure 4a). These results suggest that cell cycle arrest induced by OJE treatment might be mediated through the down‐regulation of cyclin E1/CDK2 expression and up‐regulation of p53 and p21 expression.

### 3.4 Effect of OJE on the intrinsic apoptotic and upstream signaling pathway

Apoptosis has two pathways of extrinsic (or death receptor‐dependent) and intrinsic (or mitochondrial‐dependent) factors. The intrinsic pathway is regulated by pro‐apoptotic and anti‐apoptotic proteins, such as Bax, Bak, Bid, Bcl‐2, and Bcl‐xL. In particular, the interaction between Bax and Bcl‐2 leads to a loss of mitochondrial membrane potential. As a result, mitochondrial cytochrome c is released into the cytoplasm from the mitochondria and activates the caspase pathways (Dong et al., 2014; Kassi et al., 2009). In our study, OJE‐treated cells did not exhibit dramatically altered Bax mRNA levels, but the Bcl‐2 levels were certainly decreased at the highest treatment concentration (50 μg/ml). As a result, the expression of procaspase‐3 decreased, leading to the activation of apoptosis in OVCAR‐3 cells.

Mitogen‐activated protein kinases (MAPKs) are involved in the upstream signaling pathway of various cell phenomena, such as proliferation, development, differentiation, transformation, and apoptosis. The MAPKs families consist of three subfamilies, namely ERK, JNK and p38 (Romos, 2008; Zhang & Liu, 2002). Numerous studies have focused on ERK1/2 inhibition leading to cancer cell death (Lunghi et al., 2003; Wang et al., 2010; Wu, Wong, Khosravi, Minden, & Penn, 2004). Furthermore, p38 MAPK plays a role in cell cycle arrest by activating cell cycle checkpoints (Feng et al., 2015; Thornton & Rincon, 2009). To determine the MAPK signaling pathways, we examined the activity of subfamilies such as ERK1/2 and JNK as well as p38 phosphorylation using western blot analysis. As shown in Figure 4, the expression of phosphorylated ERK1/2 (p‐ERK1/2) was significantly reduced and phosphorylated p38 (p‐p38) was gradually increased. The effective combination of p‐ERK1/2 and p‐p38 is expected to act in concert as a positive apoptosis mediator in OJE‐treated OVCAR‐3 cancer cells.

## 4 CONCLUSION

In conclusion, in our study OJE was found to produce anticancer effects via the induction of apoptosis and cell cycle arrest in OVCAR‐3 cells. Sub‐G1 cell cycle arrest induced by OJE occurred through cyclin E1/CDK2, p21, and p53‐mediated pathways. Furthermore, the down‐regulation of Bcl‐2 and pro‐caspase‐3 indicated that the apoptosis signaling pathway was mediated through mitochondria. Upstream MAPKs signaling pathways play important roles in the OJE‐induced anticancer activity observed in OVCAR‐3 cancer cells. These results suggest that OJE is expected to be used as a powerful anti‐cancer drug derived from nature in OVCAR‐3 human ovarian cancer cells.

## CONFLICT OF INTEREST

The authors declare no conflict of interest.
